# Characterization of immune responses in fully vaccinated individuals following breakthrough infection with the SARS-CoV-2 delta variant

**DOI:** 10.1126/scitranslmed.abn6150

**Published:** 2022-03-08

**Authors:** Ai-ris Y. Collier, Catherine M. Brown, Katherine A. McMahan, Jingyou Yu, Jinyan Liu, Catherine Jacob-Dolan, Abishek Chandrashekar, Dylan Tierney, Jessica L. Ansel, Marjorie Rowe, Daniel Sellers, Kunza Ahmad, Ricardo Aguayo, Tochi Anioke, Sarah Gardner, Mazuba Siamatu, Lorraine Bermudez Rivera, Michele R. Hacker, Lawrence C. Madoff, Dan H. Barouch

**Affiliations:** ^1^ Center for Virology and Vaccine Research, Beth Israel Deaconess Medical Center, Boston, MA, USA 02115; ^2^ Harvard Medical School, Boston, MA, USA 02115; ^3^ Department of Obstetrics and Gynecology, Beth Israel Deaconess Medical Center, Boston, MA, USA 02215; ^4^ Massachusetts Department of Public Health, Boston, MA, USA 02108; ^5^ Ragon Institute of MGH, MIT, and Harvard, Cambridge, MA, USA 02139

## Abstract

Breakthrough infections with severe acute respiratory syndrome coronavirus 2 (SARS-CoV-2) variants have been reported frequently in vaccinated individuals with waning immunity. In particular, a cluster of over 1,000 infections with the SARS-CoV-2 delta variant was identified in a predominantly fully vaccinated population in Provincetown, Massachusetts in July 2021. In this study, vaccinated individuals who tested positive for SARS-CoV-2 (n=16) demonstrated substantially higher serum antibody responses than vaccinated individuals who tested negative for SARS-CoV-2 (n=23), including 32-fold higher binding antibody titers and 31-fold higher neutralizing antibody titers against the SARS-CoV-2 delta variant. Vaccinated individuals who tested positive also showed higher mucosal antibody responses in nasal secretions and higher Spike protein-specific CD8^+^ T cell responses in peripheral blood than did vaccinated individuals who tested negative. These data demonstrate that fully vaccinated individuals developed robust anamnestic antibody and T cell responses following infection with the SARS-CoV-2 delta variant. Moreover, these findings suggest that population immunity will likely increase over time by a combination of widespread vaccination and breakthrough infections.

## Teaser

SARS-CoV-2 breakthrough infections led to robust serum and mucosal antibody responses and peripheral T cell responses in vaccinated individuals.

## INTRODUCTION

A large cluster of coronavirus disease 2019 (COVID-19) infections was reported to the Massachusetts Department of Public Health (MA DPH) after the July 4, 2021 weekend in Provincetown, Barnstable County, Massachusetts (*1*). Approximately 74% of cases occurred in individuals who were fully vaccinated with the BNT162b2 (Pfizer/BioNTech), mRNA-1273 (Moderna), or Ad26.COV2.S (Johnson & Johnson) COVID-19 vaccines (*2*–*4*). Most cases were reported to be mildly or moderately symptomatic. Viral sequencing revealed that over 90% of cases sequenced were the severe acute respiratory syndrome coronavirus 2 (SARS-CoV-2) delta variant, and viral loads in nasal swabs were similar in vaccinated and unvaccinated individuals (*1*). This outbreak was the first known large cluster of infections with the SARS-CoV-2 delta variant in a highly vaccinated population, and it prompted the U.S. Centers for Disease Control and Prevention (CDC) to reinstate indoor masking recommendations for fully vaccinated individuals.

Here, we recruited vaccinated individuals who were part of the MA DPH outbreak investigation or enhanced surveillance and who tested positive or negative for COVID-19 by nasopharyngeal swabs. These individuals participated in a detailed immunologic study at Beth Israel Deaconess Medical Center (BIDMC) in Boston, Massachusetts. We measured peripheral antibody responses, mucosal antibody responses, and cellular immune responses in this cohort of vaccinated individuals with or without SARS-CoV-2 breakthrough infection.

## RESULTS

### Participants

A total of 39 individuals from the MA DPH outbreak investigation (*1*) were enrolled, including 16 vaccinated individuals who tested positive for SARS-CoV-2 and 23 vaccinated individuals who tested negative for SARS-CoV-2 (**table S1**). Participants were predominantly white, and the majority were male. Vaccinated infected individuals were generally younger (median age 38 years; range 25 to 60) than vaccinated uninfected individuals (median age 61 years; range 24 to 77). Participants received the BNT162b2 (Pfizer; n=16), mRNA-1273 (Moderna; n=22), or Ad26.COV2.S (Johnson & Johnson; n=1) vaccines. Peripheral blood and nasal swab samples were obtained for immunologic assays at a median of 189 days (range 127 to 251) following first vaccine dose and a median of 34 days (range 0 to 67) following nasopharyngeal SARS-CoV-2 polymerase chain reaction (PCR) testing in the outbreak investigation. In the vaccinated infected group, 15 of 16 (94%) of individuals reported symptoms of COVID-19 infection, most commonly respiratory symptoms, fever, and loss of smell or taste, consistent with the overall outbreak investigation (*1*); all had asymptomatic or mild disease according to the National Institutes of Health (NIH) disease severity classification and none required hospitalization. Samples from a separate cohort of 18 unvaccinated, infected individuals with mild illness were also obtained for comparison (**table S2**).

### Humoral Immune Responses Following Exposure to Delta Variant

**Fig. 1.**
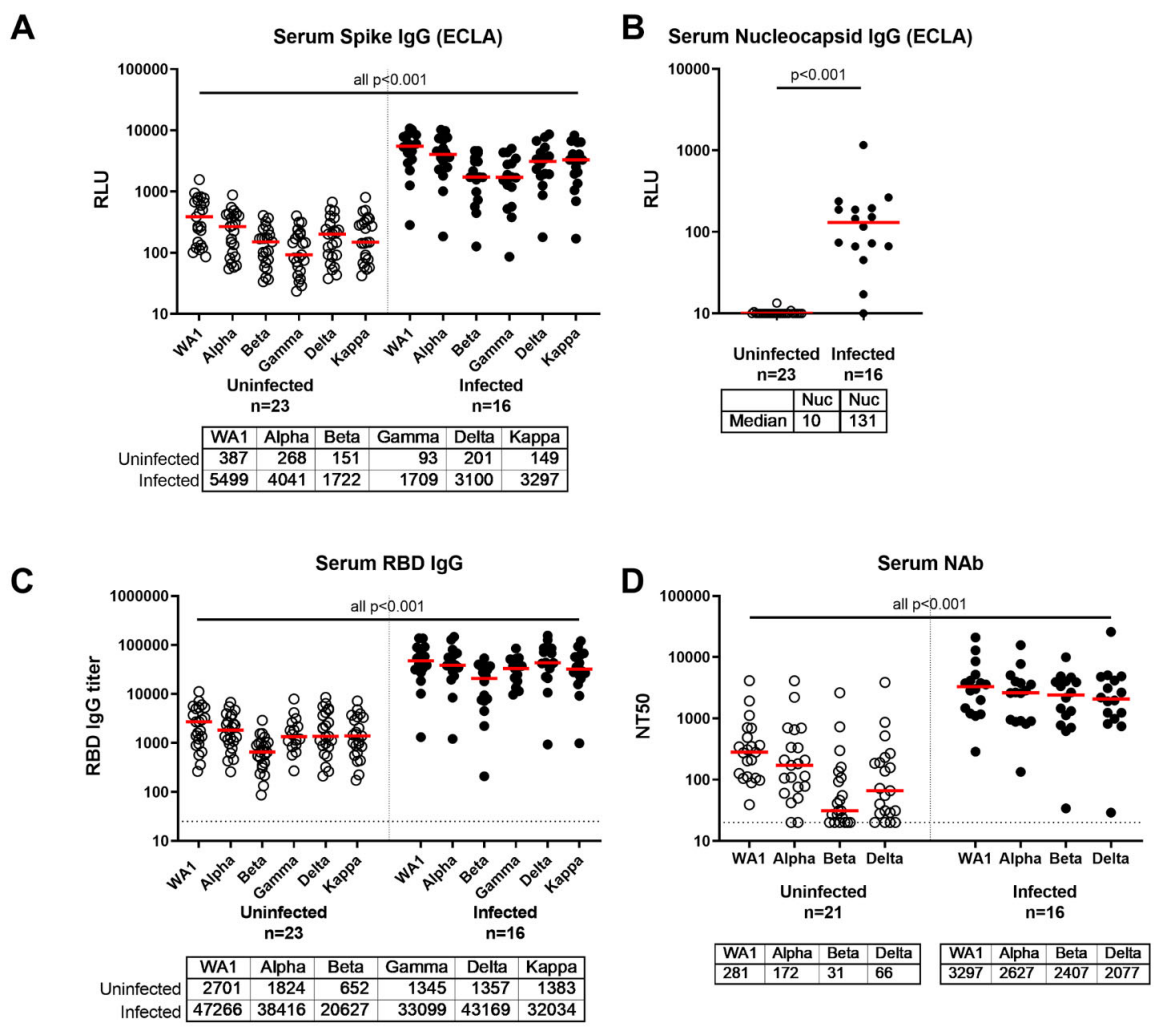
Antibody responses in COVID-19 distinguish vaccinated individuals with and without confirmed SARS-CoV-2 breakthrough infection. Serum antibody responses were measured in individuals who received BNT162b2, mRNA-1273, or Ad26.COV2.S vaccines as part of the SARS-CoV-2 outbreak investigation in Provincetown, Massachusetts. Vaccinated uninfected (open circles) and vaccinated infected (filled circles) individuals are shown. **(A and B)** IgG binding antibody titers by multiplexed electrochemiluminescence (ECLA) assays against SARS-CoV-2 Spike protein from WA1/2020, B.1.1.7 (alpha), B.1.351 (beta), P.1 (gamma), B.1.617.2 (delta), and B.1.617.1 (kappa) variants (A) and against the non-vaccine antigen Nucleocapsid (Nuc) in (B) are displayed in relative light units (RLU). **(C)** Serum IgG titers to SARS-COV-2 receptor binding domain (RBD) variants were measured by ELISA. (**D**) Pseudovirus neutralizing antibody (NAb) titers were measured in serum samples as 50% reduction (NT50) of luciferase expression. Medians (red bar) are displayed below the x-axis. Dotted horizontal lines indicate limit of detection. P values (Wilcoxon rank-sum test) compare each Spike protein variant or Nucleocapsid between uninfected and infected participants.

SARS-CoV-2 antibody responses in serum were assessed by Spike protein- and Nucleocapsid-specific electrochemiluminescence assays (ECLAs), receptor binding domain (RBD)-specific IgG enzyme-linked immunosorbent assays (ELISAs), and pseudovirus neutralizing antibody assays. Spike protein-specific ECLA titers against the SARS-CoV-2 WA1/2020, B.1.1.7 (alpha), B.1.351 (beta), P.1 (gamma), B.1.617.2 (delta), and B.1.617.1 (kappa) variants were 14-, 15-, 11-, 18-, 15-, and 22-fold higher, respectively, in vaccinated infected individuals compared with vaccinated uninfected individuals (**Fig. 1A**; P<0.001 for all variants, Wilcoxon rank-sum tests). Nucleocapsid-specific ECLA responses were detected in all but one vaccinated infected participant, consistent with SARS-CoV-2 infection, and in none of the uninfected participants (**Fig. 1B**; P<0.001, Wilcoxon rank-sum tests). RBD-specific IgG ELISA titers against the SARS-CoV-2 WA1/2020, B.1.1.7 (alpha), B.1.351 (beta), P.1 (gamma), B.1.617.2 (delta), and B.1.617.1 (kappa) variants were 17-, 21-, 31-, 25-, 32-, and 23-fold higher, respectively, in vaccinated infected individuals compared with vaccinated uninfected individuals (**Fig. 1C**; P<0.001 for all variants, Wilcoxon rank-sum tests). Pseudovirus neutralizing antibody titers against the SARS-CoV-2 WA1/2020, alpha, beta, and delta variants were 12-, 15-, 78-, and 31-fold higher, respectively, in vaccinated infected individuals compared with vaccinated uninfected individuals (**Fig. 1D**; P<0.001 for all variants, Wilcoxon rank-sum tests). Serum neutralizing antibody titers to WA1/2020 from vaccinated infected individuals were also 13-fold greater than from unvaccinated infected individuals from a separate cohort in Boston (**fig. S1A**; P<0.001, Wilcoxon rank-sum test). RBD-specific ELISA IgG titers were 46-fold greater in vaccinated infected participants compared with unvaccinated infected participants (**fig. S1B**; P<0.001, Wilcoxon rank-sum test).

Similar trends were observed in subgroup analyses of participants who received BNT162b2 or mRNA-1273 (**fig. S2**). Among vaccinated uninfected individuals, binding and neutralizing antibody responses induced by mRNA-1273 were higher than BNT162b2, and individuals who received BNT162b2 generally had low or undetectable neutralizing antibody responses against the SARS-CoV-2 delta variant at the timepoint analyzed, consistent with waning immunity (**fig. S2C**). No correlations were observed between participant age and the titers of binding and neutralizing antibodies in this cohort (**fig. S3**). Moreover, robust antibody titers were observed in vaccinated infected individuals regardless of time from vaccination (**fig. S4**). Taken together, these data demonstrate markedly higher binding and neutralizing antibody responses in vaccinated individuals who tested positive for SARS-CoV-2 after vaccination compared with vaccinated individuals who tested negative for SARS-CoV-2.

### Cellular Immune Responses Following Exposure to Delta Variant

**Fig. 2.**
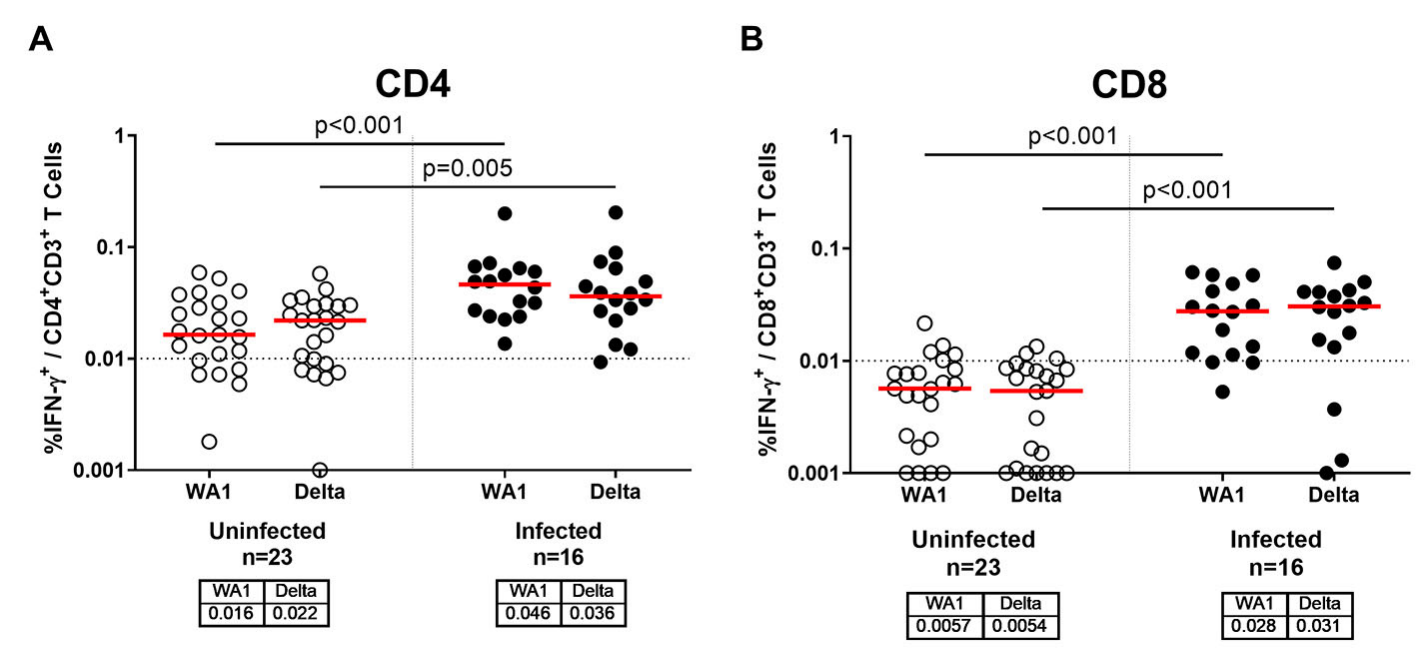
T cell responses are enhanced in COVID-19 vaccinated individuals with confirmed SARS-CoV-2 breakthrough infection compared to those without infection. (**A and B**) Intracellular cytokine staining assays were used to measure percent IFN-γ production in CD4^+^ T cells (A) and CD8^+^ T cells (B) in response to pooled Spike peptides. Vaccinated uninfected (open circles) and vaccinated infected (filled circles) individuals are shown. Assays were conducted using pooled Spike peptides from WA1/2020 or B.1.617.2 (delta). Medians (red bar) for each variant are displayed below the x-axis. Dotted horizontal lines indicates lower limit of quantitation. P values reflect Wilcoxon rank-sum tests.

SARS-CoV-2 cellular immune responses were assessed by intracellular cytokine staining assays and ELISPOT assays using pooled Spike peptides. Spike protein-specific CD4^+^ T cell responses to SARS-CoV-2 WA1/2020 and B.1.617.2 (delta) were 2.9- and 1.6-fold higher, respectively, in vaccinated infected individuals compared with vaccinated uninfected individuals (**Fig. 2A**; P<0.001 and P=0.005, respectively, Wilcoxon rank-sum tests). Median Spike protein-specific CD8^+^ T cell responses to SARS-CoV-2 WA1/2020 and B.1.617.2 (delta) were below the limit of detection in vaccinated uninfected individuals but were at least 4.9- and 5.7-fold higher, respectively, in vaccinated infected individuals (**Fig. 2B**; both P<0.001, Wilcoxon rank-sum tests). Similar trends were observed for ELISPOT responses and when CD4^+^ and CD8^+^ T cell responses were compared between individuals who received the BNT162b2 or mRNA-1273 vaccines (**fig. S5**).

### Mucosal Immune Responses Following Exposure to Delta Variant

**Fig. 3.**
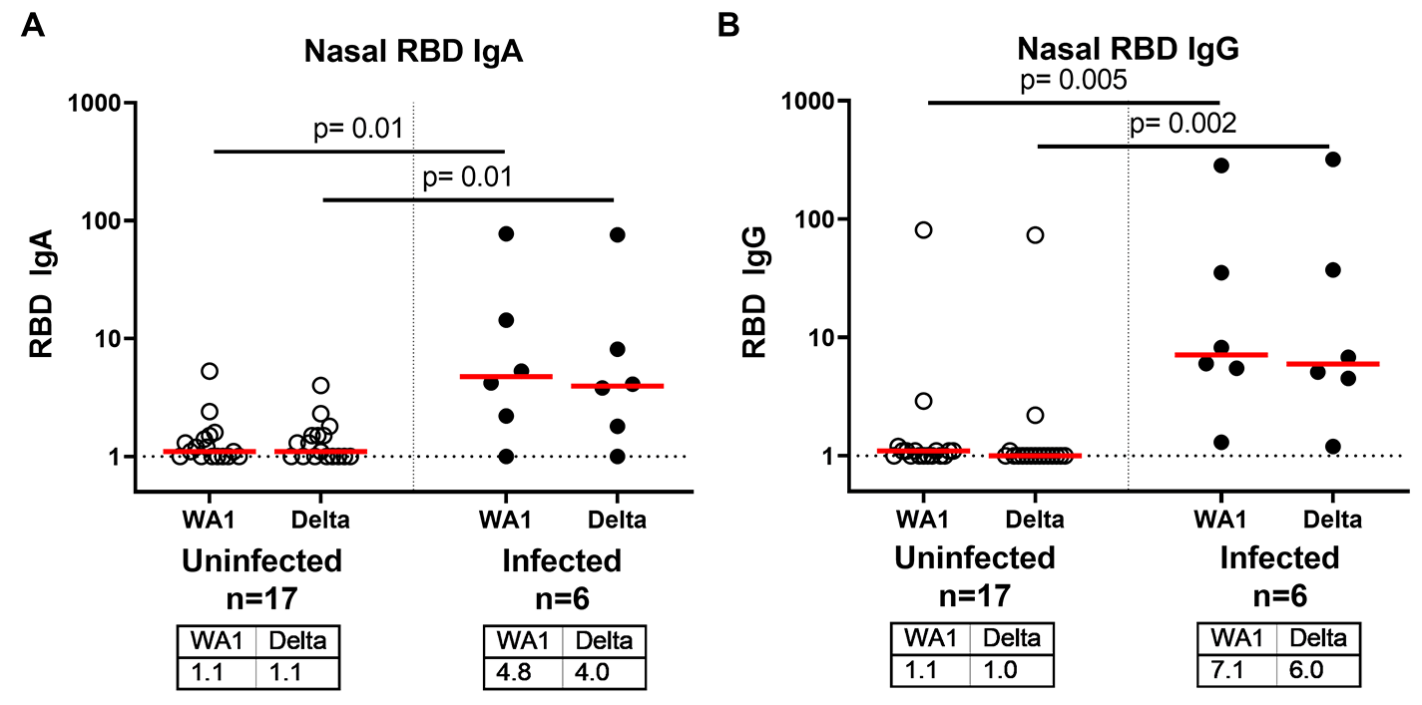
Mucosal RBD-specific nasal swab IgA and IgG responses are increased following breakthrough infection after COVID-19 vaccination compared to uninfected, vaccinated individuals. (**A and B**) SARS-CoV-2 RBD-specific IgA (A) and IgG (B) responses in nasal swabs were measured by ELISA. Vaccinated uninfected (open circles) and vaccinated infected (filled circles) individuals are shown. Nasal ELISA titers are shown against WA1/2020 and B.1.617.2 (delta) variant RBD proteins. Medians (red bar) for each variant are displayed below the x-axis. Dotted horizontal lines indicate limit of detection. P values reflect Wilcoxon rank-sum tests.

Antibody responses in the nasal mucosa may be critical for protection against respiratory viruses. In a subset of 23 individuals from whom nasal swabs were available, we assessed RBD-specific IgA and IgG responses in mucosal secretions extracted from nasal swabs. Nasal RBD-specific IgA responses to SARS-CoV-2 WA1/2020 and B.1.617.2 (delta) were 4-fold higher in vaccinated infected individuals compared with vaccinated uninfected individuals (**Fig. 3A**; p=0.01 for both, Wilcoxon rank-sum tests). Nasal RBD-specific IgG responses to SARS-CoV-2 WA1/2020 and B.1.617.2 (delta) were 6-fold higher in vaccinated infected individuals compared with vaccinated uninfected individuals (**Fig. 3B**; P=0.005 and P=0.002, respectively, Wilcoxon rank-sum tests). These data demonstrate that mucosal antibody responses induced by vaccination were low or undetectable among uninfected participants, but were substantially higher among vaccinated participants who tested positive for SARS-CoV-2.

## DISCUSSION

The increasing numbers of breakthrough infections in fully vaccinated individuals with waning immunity suggest that current SARS-CoV-2 vaccines do not provide durable sterilizing immunity, particularly against viral variants with enhanced transmissibility and reduced neutralization sensitivity. The cluster of COVID-19 infections in Provincetown, Massachusetts in July 2021 represented the first large, well-characterized outbreak of the SARS-CoV-2 delta variant in a highly vaccinated population in the United States (*1*). Vaccinated infected individuals exhibited markedly higher serum and mucosal antibody responses as well as higher cellular immune responses as compared to vaccinated uninfected individuals, suggesting that breakthrough infections triggered robust anamnestic immune responses, including against the SARS-CoV-2 delta variant. These data demonstrate a benefit of vaccination in the context of SARS-CoV-2 breakthrough infections. One BNT162b2-vaccinated individual with asymptomatic infection had low Spike protein-specific and no Nucleocapsid-specific antibody responses, but exhibited high CD4 and CD8 responses 12 days after the positive test.

Recent reports have suggested declining efficacy of the BNT162b2 vaccine after 4 to 6 months (*5*, *6*), although protection against severe disease has remained high (*7*, *8*). In the present study, participants were vaccinated a median of 5 to 6 months prior to the outbreak and thus were in the timeframe of declining antibody titers (*9*, *10*). In fact, 44% (4 of 9) of BNT162b2 vaccinated uninfected individuals had undetectable neutralizing antibody titers against the SARS-CoV-2 delta variant at the timepoint analyzed. These immunologic parameters, coupled with the high transmissibility of the delta variant and with large community gatherings in Provincetown during and after the July 4^th^ weekend may have contributed to the large cluster of breakthrough infections observed in this outbreak.

We speculate that the robust anamnestic immune responses following breakthrough SARS-CoV-2 infections in vaccinated individuals likely contribute to the low risk of severe clinical disease in people who are fully vaccinated. Consistent with these observations are data from nonhuman primate studies in which vaccinated macaques that were not fully protected following SARS-CoV-2 challenge similarly developed high anamnestic antibody responses and rapid resolution of viral replication in the upper and lower respiratory tract (*11*, *12*). We also speculate that the extraordinarily high antibody titers observed in vaccinated individuals who develop breakthrough infections may lead to subsequent long-term protection in those individuals.

One limitation of this study is the relatively small number of individuals in this immunologic analysis compared with the large total number of individuals in this outbreak. We only had one Ad26.COV2.S-vaccinated individual in this study, which limits the ability to compare immune responses across the three available vaccines. Nevertheless, the magnitude and consistency of the immunologic differences observed between vaccinated infected and vaccinated uninfected individuals suggest the generalizability of the conclusions. Another limitation is the fact that the vaccinated infected group was generally younger than the vaccinated uninfected group, which may reflect different risk behaviors or exposures based on age. In addition, based on the Massachusetts prioritization of vaccine administration, older individuals in this cohort may have had a longer interval from vaccination to exposure to the SARS-CoV-2 delta variant with associated waning vaccine immunity. Individuals less than 65 years old were vaccinated 127 to 251 days prior, similar to 175 to 236 days prior in those over 65 years old. Overall, age did not appear to correlate with the magnitude of binding or neutralizing antibody titers in this cohort.

In conclusion, we describe humoral and cellular immune responses in a large, well described cluster of breakthrough infections with the SARS-CoV-2 delta variant in fully vaccinated individuals in the United States. Breakthrough infections led to increases in serum and mucosal antibody responses and peripheral T cell responses in vaccinated individuals. Anamnestic antibody responses in breakthrough infections were similar in magnitude regardless of the length of time from vaccination, suggesting the possibility of protection against severe disease for a prolonged period of time, possibly even after serum antibody titers decline. These data provide insights into the immunology of breakthrough infections with the highly transmissible SARS-CoV-2 delta variant in fully vaccinated populations. Moreover, these findings suggest that population immunity will likely increase over time by a combination of widespread vaccination and breakthrough infections with SARS-CoV-2 variants.

## MATERIALS AND METHODS

### Study design

We conducted an exploratory, descriptive cohort study of individuals ≥18 years of age who were part of the outbreak investigation of the breakthrough infections in Provincetown, MA. The MA Department of Public Health and Boston Public Health Commission provided information about this immunologic study to individuals by telephone or email. Participants were provided contact information for the Beth Israel Deaconess Medical Center (BIDMC) study team for recruitment and informed consent. Participants also self-referred from flyers posted on social media. Participants were asked to provide their vaccine, symptom, and testing history, as well as their race and ethnicity based on specified categories; they could select multiple race categories. The BIDMC Institutional Review Board (IRB) approved this study (#2021P000344) as part of a parent biorepository study (#2020P000361). All participants provided informed consent. Breakthrough SARS-CoV-2 infection was defined as a positive nasopharyngeal swab PCR test after being fully vaccinated (at least 2 weeks following two doses of the BNT162b2 or mRNA-1273 vaccines or at least 2 weeks after a single dose of the Ad26.COV2.S vaccine). Participants were asked to provide blood samples and nasal swabs. Participants were excluded if they had past infection with SARS-CoV-2 prior to vaccination or received an additional booster dose of vaccine during the study period. Participants were enrolled as a convenience sample. No a priori power analysis was performed and no outliers were excluded. We also obtained samples (n=18) from unvaccinated participants in Boston with SARS-CoV-2 infection between March to November 2020 as part of the parent biorepository study (#2020P000361). The majority of these participants had asymptomatic or mild illness (78%, n=14). Assay operators were all blinded to the participant prior infection or vaccination history.

### Electrochemiluminescence assay (ECLA)

ECLA plates (MesoScale Discovery, MSD; SARS-CoV-2 IgG Cat No: N05CA-1; Panel 7 and K15463U-2; Panel 13) were designed and produced with up to 9 antigen spots in each well, and assays were performed essentially as described previously (*13*). The antigens included Spike proteins from WA1/2020, B.1.1.7 (alpha), B.1.351 (beta), P.1 (gamma), B.1.617.2 (delta), and B.1.617.1 (kappa) as well as WA1/2020 Nucleocapsid (Nuc). The plates were blocked with 50 μL of Blocker A (1% bovine serum albumin in distilled water) solution for at least 30 min at room temperature with shaking at 700 rpm with a digital microplate shaker. During blocking, the serum was diluted to 1:5,000 in Diluent 100. The calibrator curve was prepared by diluting the calibrator mixture from MSD 1:9 in Diluent 100 and then preparing a 7-step 4-fold dilution series plus a blank containing only Diluent 100. The plates were then washed 3 times with 150 μL of Wash Buffer (0.5% Tween in 1x phosphate-buffered saline [PBS]), blotted dry, and 50 μL of the diluted samples and calibration curve were added in duplicate to the plates and set to shake at 700 rpm at room temperature for at least 2 hours. The plates were again washed 3 times and 50 μL of SULFO-Tagged anti-Human IgG detection antibody diluted to 1x in Diluent 100 was added to each well. Samples were incubated with shaking at 700 rpm at room temperature for at least 1 hour. Plates were then washed 3 times and 150 μL of MSD GOLD Read Buffer B was added to each well. The plates were read immediately after on a MESO QuickPlex SQ 120 machine. MSD titers for each sample were reported as relative light units (RLU), which were calculated using the calibrator.

### Enzyme-linked immunosorbent assay (ELISA)

SARS-CoV-2 Spike protein RBD-specific binding antibodies in serum were assessed by ELISA as described previously (*13*). 96-well plates were coated with 2 μg/mL of SARS-CoV-2 WA1/2020, Alpha, Beta, Gamma, Delta, or Kappa RBD protein (provided by F. Krammer, Icahn School of Medicine at Mount Sinai) in 1× Dulbecco’s phosphate-buffered saline (DPBS) and incubated at 4°C overnight. After incubation, plates were washed once with wash buffer (0.05% Tween 20 in 1× DPBS) and blocked with 350 μL of casein block solution per well for 2 to 3 hours at room temperature. Following incubation, block solution was discarded and plates were blotted dry. Serial dilutions of heat-inactivated serum diluted in Casein block or nasal swab sample diluted in DPBS were added to wells, and plates were incubated for 1 hour at room temperature prior to 3 more washes. Samples were then incubated for 1 hour with a 1:4000 dilution of anti–human IgG horseradish peroxidase (HRP, Invitrogen) or 1:1000 dilution of anti-human IgA HRP (Bethyl Laboratories) at room temperature in the dark. Plates were washed 3 times, and 100 μL of SeraCare KPL 3,3′,5,5′-Tetramethylbenzidine (TMB) SureBlue Start solution was added to each well; plate development was halted by adding 100 μL of SeraCare KPL TMB Stop solution per well. The absorbance at 450 nm, with a reference at 650 nm, was recorded with a VersaMax microplate reader (Molecular Devices). For each sample, the ELISA end point titer was calculated using a 4-parameter logistic curve fit to calculate the reciprocal serum dilution that yields a corrected absorbance value (450 nm-650 nm) of 0.2. Interpolated end point titers were reported.

### Pseudovirus neutralizing antibody assay

SARS-CoV-2 pseudoviruses expressing a luciferase reporter gene were used to measure pseudovirus neutralizing antibodies as described previously (*13*, *14*). In brief, the packaging construct psPAX2 (AIDS Resource and Reagent Program), luciferase reporter plasmid pLenti-CMV Puro-Luc (Addgene) and Spike protein expressing pcDNA3.1-SARS-CoV-2 SΔCT were co-transfected into HEK293T cells (American Type Culture Collection [ATCC] CRL_3216) with lipofectamine 2000 (Thermo Fisher Scientific). Pseudoviruses of SARS-CoV-2 variants were generated by using WA1/2020 strain (Wuhan/WIV04/2019, GISAID accession ID: EPI_ISL_402124), B.1.1.7 variant (Alpha, GISAID accession ID: EPI_ISL_601443), B.1.351 variant (Beta, GISAID accession ID: EPI_ISL_712096), or B.1.617.2 (Delta, GISAID accession ID: EPI_ISL_2020950). The supernatants containing the pseudotype viruses were collected 48 hours after transfection and pseudotype viruses were purified by filtration with 0.45 μm filter. To determine the neutralization activity of human serum, HEK293T cells expressing human angiotensin converting enzyme 2 (HEK293T-hACE2 cells) were seeded in 96-well tissue culture plates at a density of 1.75 × 10^4^ cells per well overnight. Three-fold serial dilutions of heat-inactivated serum samples were prepared and mixed with 50 μl of pseudovirus. The mixture was incubated at 37°C for 1 hour before adding to HEK293T-hACE2 cells. After 48 hours, cells were lysed in a Steady-Glo Luciferase Assay (Promega) according to the manufacturer’s instructions. SARS-CoV-2 neutralization titers were defined as the sample dilution at which a 50% reduction (NT50) in relative light units was observed relative to the average of the virus control wells. Serum samples with dense lipophilic debris after heat inactivation were excluded from the pseudovirus neutralization analysis due to the inability to acquire an accurate measure of relative light units.

### Intracellular cytokine staining assays

10^6^ peripheral blood mononuclear cells well were re-suspended in 100 μL of R10 media (RPMI-1640 with 10% heat inactivated fetal bovine serum, 1% of 100x penicillin-streptomycin, 1M HEPES, 100mM Sodium pyruvate, 200mM L-glutamine, and 0.1% of 55mM 2-Mercaptoethanol) supplemented with CD49d monoclonal antibody (1 μg/mL, BD Biosciences) and CD28 monoclonal antibody (1 μg/mL, BD Biosciences) as described previously (*13*). Each sample was assessed with mock (100 μL of R10 plus 0.5% dimethyl sulfoxide; background control), peptides (2 μg/mL), or 10 pg/mL phorbol myristate acetate (PMA, Sigma-Aldrich) and 1 μg/mL ionomycin (Sigma-Aldrich) (100 μL; positive control) and incubated at 37°C for 1 hour. After incubation, 0.25 μL of GolgiStop and 0.25 μL of GolgiPlug in 50 μL of R10 was added to each well and incubated at 37°C for 8 hours and then held at 4°C overnight. The next day, the cells were washed twice with DPBS, stained with aqua live/dead dye (Life Technologies) for 10 min and then stained with monoclonal antibodies against CD279 (clone EH12.1, Brilliant Blue 700), CD4 (clone L200, Brilliant Violet 711), CD27 (clone M-T271, Brilliant Ultraviolet (BUV) 563), CD8 (clone SK1, BUV805), CD45RA (clone 5H9, Allophycocyanin (APC) H7) for 30 min. Cells were then washed twice with 2% fetal bovine serum in PBS buffer and incubated for 15 min with 200 μL of BD CytoFix/CytoPerm Fixation/Permeabilization solution. Cells were washed twice with 1X Perm Wash buffer (BD Perm/Wash Buffer 10X in the CytoFix/CytoPerm Fixation/ Permeabilization kit diluted with MilliQ water and passed through 0.22μm filter) and stained with intracellularly with monoclonal antibodies against interferon (IFN)-γ (clone B27; BUV395), and CD3 (clone SP34.2, Alexa Fluor 700) for 30 min. Cells were washed twice with 1X Perm Wash buffer and fixed with 250μL of freshly prepared 1.5% formaldehyde. Fixed cells were transferred to 96-well round bottom plate and analyzed by BD FACSymphony system. Data were analyzed using FlowJo v9.9.

### Enzyme-linked immunospot (ELISPOT) assay

ELISPOT plates were coated with mouse anti-human IFN-γ monoclonal antibody from MabTech at 1 μg/well and incubated overnight at 4°C. Plates were washed with DPBS, and blocked with R10 media for 2 to 4 hours at 37°C. SARS-CoV-2 pooled S peptides from WA1/2020, B.1.351, B.1.1.7, and B.1.617.2 (21st Century Biochemicals) were prepared and plated at a concentration of 2 μg/well, and 100,000 cells per well were added to the plate. The peptides and cells were incubated for 15 to 20 hours at 37°C. All steps following this incubation were performed at room temperature. The plates were washed with ELISPOT wash buffer and incubated for 2 to 4 hours with biotinylated mouse anti-human IFN-γ monoclonal antibody from MabTech (1 μg/mL). The plates were washed a second time and incubated for 2 to 3 hours with conjugated goat anti-biotin alkaline phosphatase from Rockland, Inc. (1.33 μg/mL). The final wash was followed by the addition of Nitro-blue Tetrazolium Chloride/5-bromo-4-chloro 3 ‘indolyphosphate p-toludine salt (NBT/BCIP chromagen) substrate solution for 7 min. The chromagen was discarded and the plates were washed with water and dried in a dim place for 24 hours. Plates were scanned and counted on a Cellular Technologies Limited Immunospot Analyzer.

### Statistical analysis

Descriptive statistics were calculated using GraphPad Prism 8.4.3. All data points represent biological replicates for each group. Each immunologic assay was performed for each sample independently without technical replicates. Data are presented as median with interquartile range (IQR). Comparisons were performed using the Wilcoxon rank-sum test. Spearman correlation coefficient (r) was reported for antibody titers with time from first vaccine dose and age. All tests were two-tailed. When all six variants were compared between infected and uninfected or between BNT162b2 and mRNA-1273, a p value < 0.01 was considered statistically significant to account for multiple comparisons. For all other comparisons, p values < 0.05 were considered statistically significant.

## Supplementary Materials

### Other Supplementary Material for this manuscript includes the following:

MDAR Reproducibility Checklist
