# Supplementary material for: Characterization of immune responses in fully vaccinated individuals following breakthrough infection with the SARS-CoV-2 delta variant
Source: Sci Transl Med. 2022 Mar 8:eabn6150. doi: 10.1126/scitranslmed.abn6150 (PMC8995036; doi:10.1126/scitranslmed.abn6150)
Supplement: Supplementary file 1 — Figs. S1 to S5 Tables S1 and S2 [file scitranslmed.abn6150_sm.pdf]

Science Translational Medicine
AAAS

Supplementary Materials for

Characterization of immune responses in fully vaccinated individuals following breakthrough infection with the SARS-CoV-2 delta variant

Ai-ris Y. Collier *et al.*

Corresponding author: Dan H. Barouch, dbarouch@bidmc.harvard.edu

DOI: 10.1126/scitranslmed.abn6150

The PDF file includes:

Figs. S1 to S5
Tables S1 and S2

Other Supplementary Material for this manuscript includes the following:

MDAR Reproducibility Checklist
Data file S1

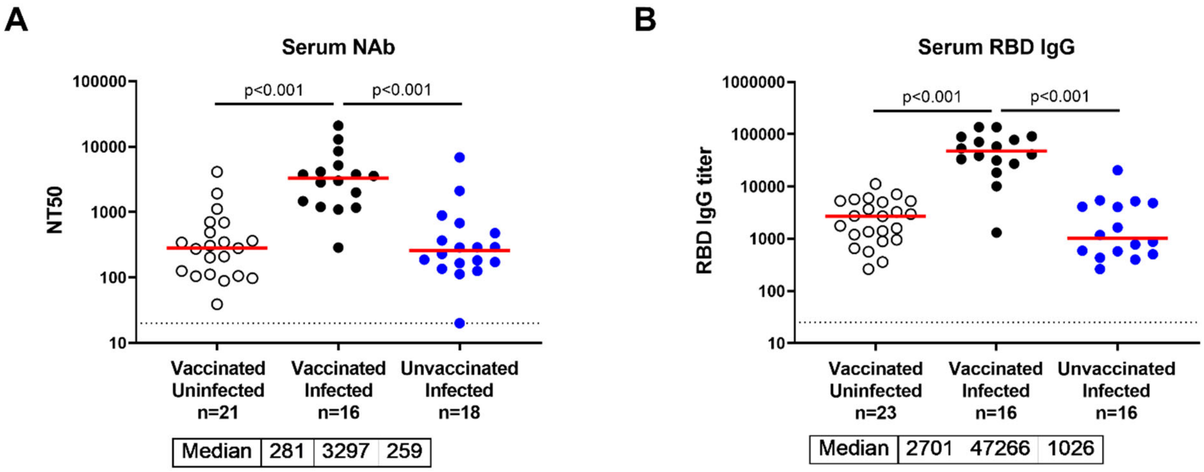

Fig. S1. Antibody responses in coronavirus disease 2019 (COVID-19) vaccinated individuals and unvaccinated individuals with severe acute respiratory syndrome coronavirus 2 (SARS-CoV-2) infection.

Serum antibody responses in vaccinated individuals exposed to the SARS-CoV-2 outbreak in Provincetown, Massachusetts (presented in **Fig. 1** of the main text) were compared directly with an unvaccinated early pandemic cohort from Boston. Vaccinated uninfected individuals (open symbols), vaccinated infected (filled black symbols) individuals, and unvaccinated individuals with SARS-CoV-2 infection from the early pandemic (filled blue symbols) are shown. (**A**) Pseudovirus neutralization titers were measured as 50% reduction (NT50) of luciferase expression for the Spike WA1/2020 variant. NAb, neutralizing antibody. (**B**) Enzyme-linked immunosorbent assay (ELISA) IgG titers are shown for the SARS-COV-2 receptor binding domain (RBD) for the WA1/2020 variant. Medians (red bar) for each variant are displayed below the x-axis. The dotted line indicates limit of detection. P values compare two-way unpaired medians titer (Wilcoxon rank-sum test).

A

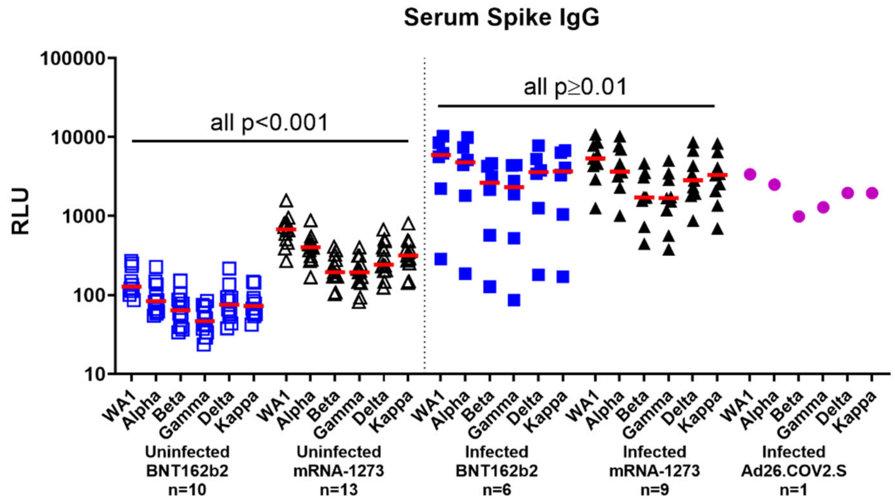

	WA1	Alpha	Beta	Gamma	Delta	Kappa
BNT162b2	127	84	64	46	75	72
mRNA-1273	675	403	195	194	242	316

	WA1	Alpha	Beta	Gamma	Delta	Kappa
	5924	4782	2638	2327	3594	3682
	5352	3644	1716	1682	2823	3300
Ad26.COV2.S	3369	2492	987	1287	1958	1947

B

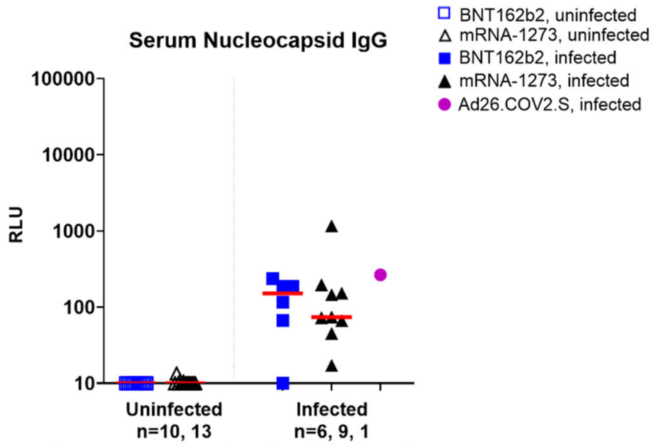

	Nuc	Nuc
Median	10	10

Nuc	Nuc	Nuc
152	74	265

C

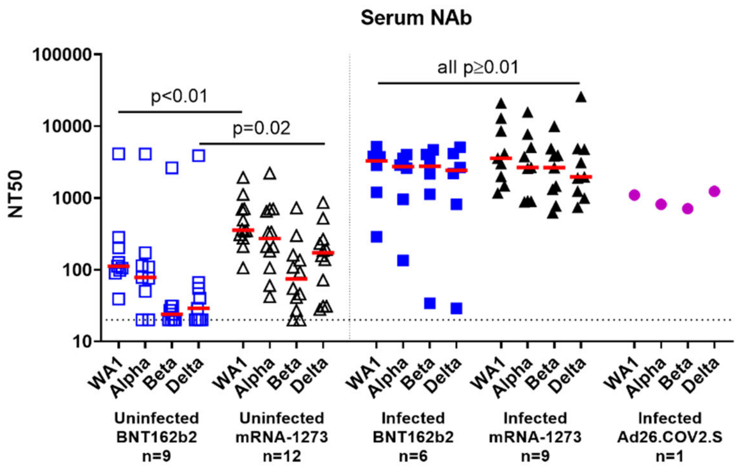

	WA1	Alpha	Beta	Delta
BNT162b2	112	78	24	29
mRNA-1273	357	271	74	171

	WA1	Alpha	Beta	Delta
	3294	2730	2757	2421
	3578	2655	2646	1965
Ad26.COV2.S	1099	812	707	1234

D

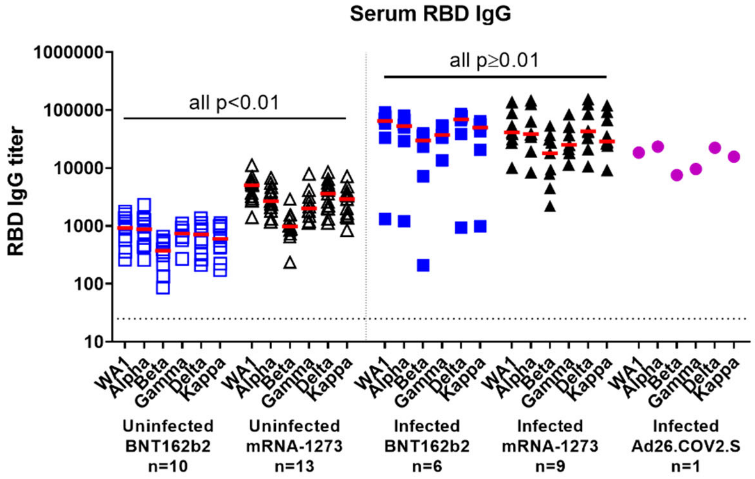

	WA1	Alpha	Beta	Gamma	Delta	Kappa
BNT162b2	924	871	374	738	710	595
mRNA-1273	5012	2675	982	1996	3609	2897

	WA1	Alpha	Beta	Gamma	Delta	Kappa
	64517	52681	29681	37136	68954	49793
	41167	38361	18085	25018	42759	28764
Ad26.COV2.S	18394	23337	7526	9593	22293	15606

Fig. S2. Antibody responses in COVID-19 vaccinated individuals with and without confirmed SARS-CoV-2 breakthrough infection, by vaccine type.

Serum antibody responses were measured in samples from individuals vaccinated with BNT162b2 (blue), mRNA-1273 (black), or Ad26.COV2.S (magenta) as part of the SARS-CoV-2 outbreak investigation in Provincetown, Massachusetts. Vaccinated uninfected (open symbols) and vaccinated infected (filled symbols) individuals are shown. (**A** and **B**) IgG binding antibody titers measured by multiplexed electrochemiluminescence (ECLA) assays against SARS-CoV-2 Spike from WA1/2020, B.1.1.7 (alpha), B.1.351 (beta), P.1 (gamma), B.1.617.2 (delta), and B.1.617.1 (kappa) variants (A) and against the non-vaccine antigen Nucleocapsid (Nuc) (B) are displayed in relative light units (RLU). (**C**) Pseudovirus neutralization titers were measured as 50% reduction (NT50) of luciferase expression. (**D**) ELISA IgG titers to SARS-COV-2 RBD variants are shown. For all panels, median values (red bar) for each variant are displayed below the x-axis. Dotted line indicates limit of detection. P values (Wilcoxon rank-sum test) compare the Spike protein or Nucleocapsid from the indicated variants between participants who received BNT162b2 and those who received mRNA-1273.

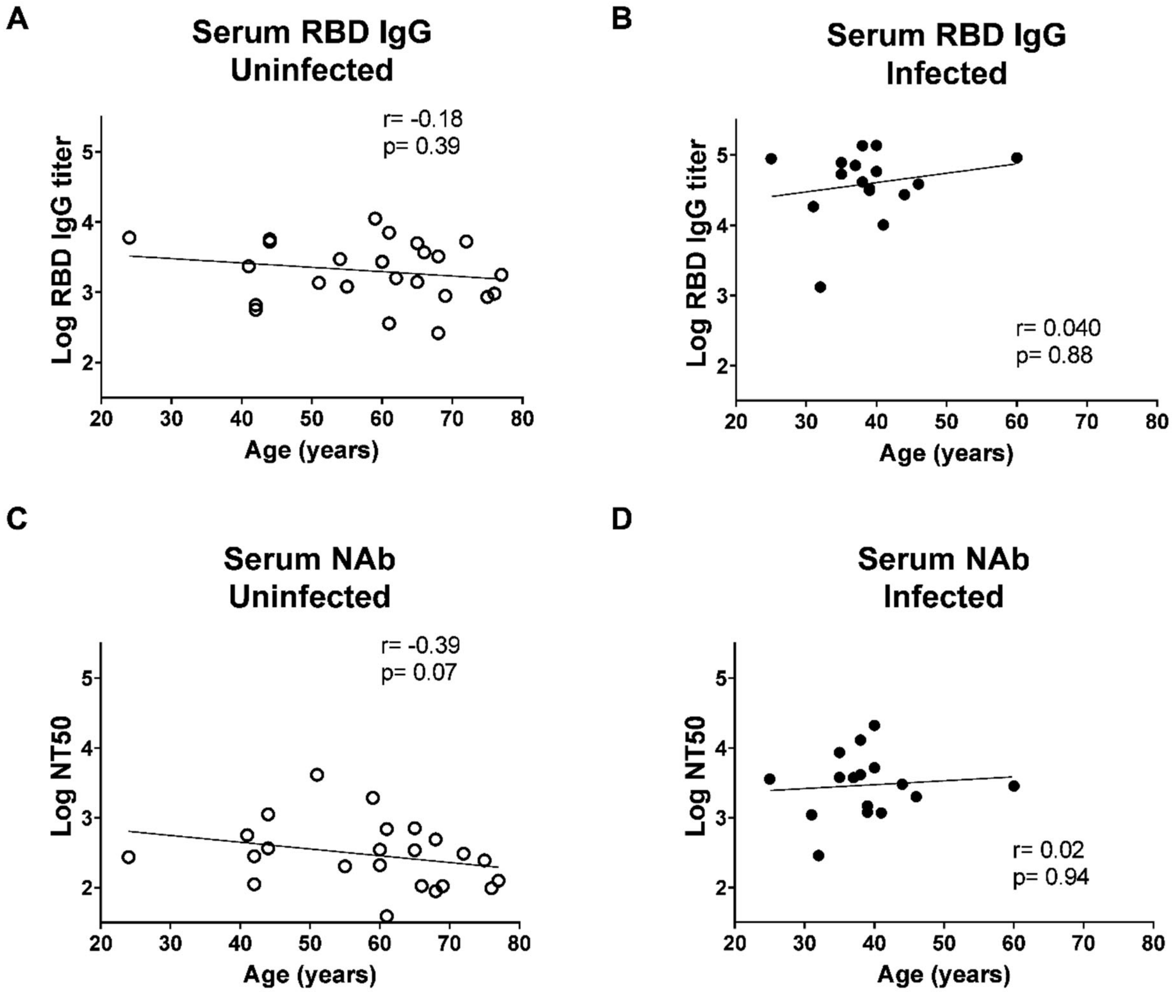

Fig. S3. RBD-binding serum IgG titer and neutralizing antibody titer by age.

Serum RBD-specific ELISA IgG titers (**A and B**) and pseudovirus NT50 NAb titers (**C and D**) against WA1/2020 are plotted as a function of age. Vaccinated uninfected (**A, C**) and vaccinated infected (**B, D**) individuals are shown with line of best fit. Spearman correlation (r) and p-values are listed for each plot.

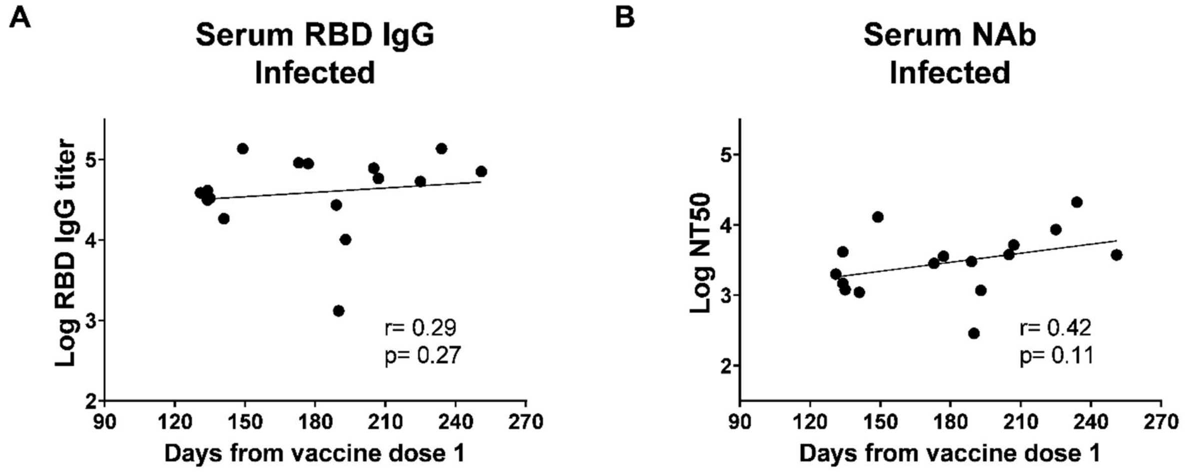

Fig. S4. Serum RBD-binding IgG titer and neutralizing antibody in vaccinated and infected individuals by days from first COVID-19 vaccine dose.

(**A and B**) Serum RBD-specific ELISA IgG titer (A) and pseudovirus NT50 neutralizing antibody (NAb) titer (B) against WA1/2020 are plotted against days from first COVID-19 vaccine dose in vaccinated and infected individuals. Data are shown with a line of best fit. Spearman correlation (r) and p-values are listed for each plot.

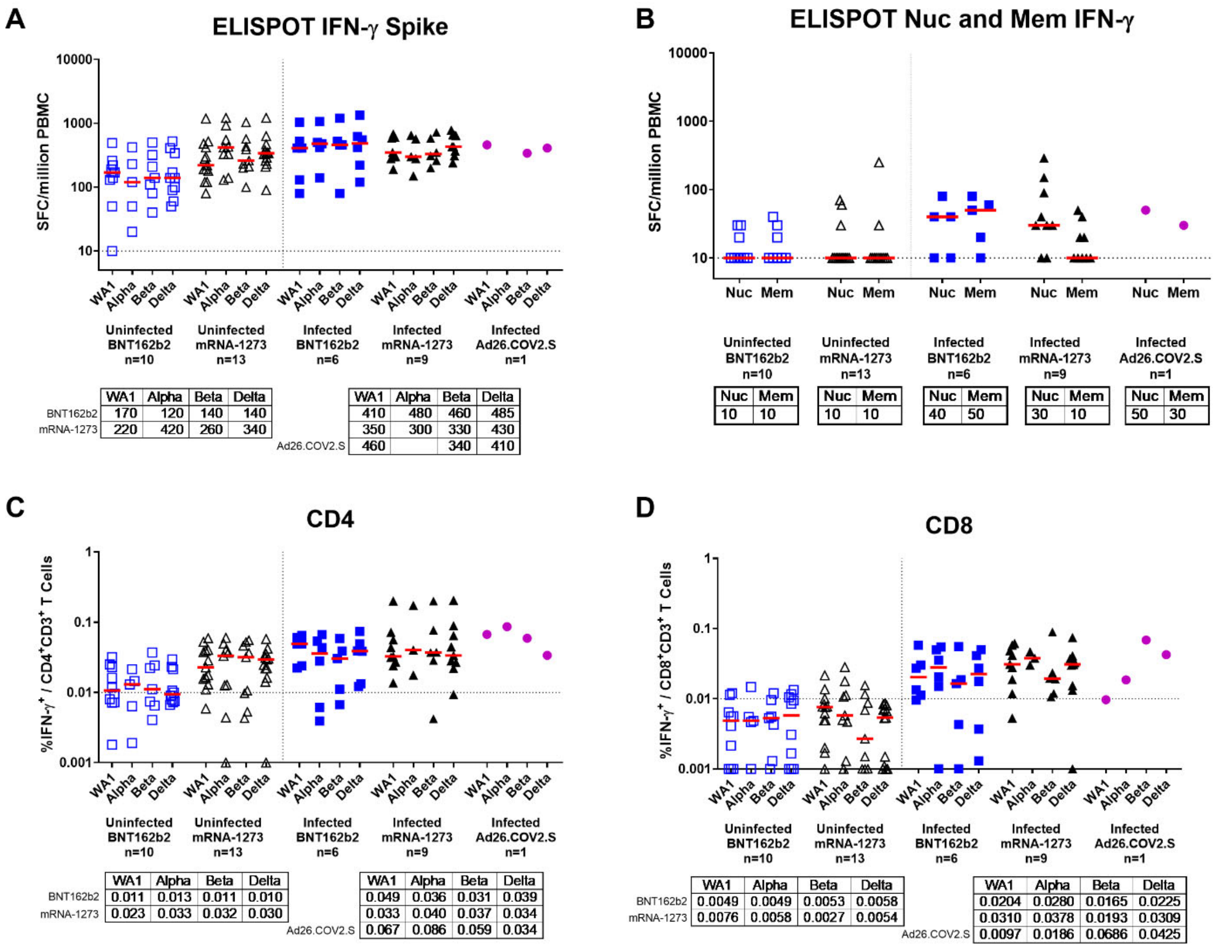

Fig. S5. T cell responses in COVID-19 vaccinated individuals with and without confirmed SARS-CoV-2 breakthrough infection, by vaccine type.

(**A and B**) SARS-CoV-2-specific T cell responses in BNT162b2 (blue), mRNA-1273 (black), and Ad26.COV2.S (magenta) vaccinated individuals were measured by interferon (IFN)-γ enzyme-linked immune absorbent spot (ELISPOT) assays after stimulation with pooled Spike peptides from WA1/2020 or from variants B.1.1.7 (alpha), B.1.351 (beta), and B.1.617.2 (delta) (A) or non-vaccine antigens Nucleocapsid (Nuc) and Membrane (Mem) (B). Data are presented as spot-forming cells (SFCs) per million peripheral blood mononuclear cells (PBMCs). (**C and D**) Intracellular cytokine staining (ICS) assays measured percent IFN-γ production in CD4 T cells (C)

and CD8 T cells (D) in response to Spike peptides from indicated variants. Vaccinated uninfected (open symbols) and vaccinated infected (filled symbols) individuals are shown. Medians (red bar) for each variant are displayed below the x-axis. Dotted line indicates limit of detection for (A) and (B) and indicates the lower limit of quantitation for (C) and (D).

Table S1. Characteristics of vaccinated study population

	Vaccinated, infected n=16	Vaccinated, uninfected n=23
Age (years), median (IQR)	38 (35 to 40)	61 (52 to 67)
Sex at birth, female, n (%)	3 (19)	8 (35)
Race, n (%)		
White	13 (81)	23 (100)
Black	0	0
Asian	1 (6)	0
More than one race	0	0
Another race	1 (6)	0
Ethnicity, n (%)		
Hispanic or Latino	0	1 (4)
Non-Hispanic	16 (100)	22 (96)
Medical condition, n (%)		
Pregnant	0	0
Diabetes	0	2 (9)
Hypertension	3 (19)	7 (30)
Obesity (Body mass index ≥ 30 kg/m^2)	3 (19)	2 (9)
Asthma	3 (19)	4 (17)
COVID19 Vaccine, n (%)		
BNT162b2	6 (38)	10 (44)
mRNA-1273	9 (56)	13 (57)
Ad26.COV2.S	1 (6)	0
Days from 1st vaccine dose to sampling, median (IQR)	183 (137 to 207)	191 (158 to 221)
Days from PCR test to sampling, median (IQR)	33 (22 to 42)	35 (29 to 47)
Symptoms, n	15	14
Never symptomatic, n (%)	0	8 (57)
Respiratory, n (%)	14 (93)	6 (43)
Gastrointestinal, n (%)	2 (13)	0
Fever, n (%)	8 (53)	0
Sore throat, n (%)	7 (47)	1 (7)
Loss of smell or taste, n (%)	13 (87)	0
Other, n (%)	9 (60)	4 (29)
NIH disease severity, n (%)		
Asymptomatic	1 (6)	N/A
Mild/Moderate	15 (94)	N/A
Severe/Critical	0	N/A

IQR, interquartile range; PCR= polymerase chain reaction nasopharyngeal testing for SARS-CoV-2; NIH, National Institutes for Health; N/A, not applicable.

Table S2. Characteristics of unvaccinated cohort

	Unvaccinated, infected n=18
Age (years), median (IQR)	38 (33-43)
Sex at birth, female, n (%)	16 (89)
Race, n (%)	
White	8 (44)
Black	5 (28)
Asian	0
More than one race	0
Another race	5 (18)
Ethnicity, n (%)	
Hispanic or Latino	2 (11)
Non-Hispanic	13 (72)
Unknown	3 (17)
Medical condition, n (%)	
Pregnant	10 (56)
Diabetes	1 (6)
Hypertension	2 (11)
Obesity (Body mass index ≥ 30 kg/m^2)	8 (44)
Asthma	2 (11)
Days from PCR test to sampling, median (IQR)	27 (19-52)
NIH disease severity, n (%)	
Asymptomatic	1 (6)
Mild/Moderate	14 (78)
Severe	3 (17)
